# A comparison of principal component analysis, partial least-squares, and reduced-rank regressions in the identification of dietary patterns associated with hypertension: YaHS-TAMYZ and Shahedieh cohort studies

**DOI:** 10.3389/fnut.2022.1076723

**Published:** 2023-01-12

**Authors:** Sara Beigrezaei, Sara Jambarsang, Sayyed Saeid Khayyatzadeh, Masoud Mirzaei, Amir Houshang Mehrparvar, Amin Salehi-Abargouei

**Affiliations:** ^1^Nutrition and Food Security Research Center, Shahid Sadoughi University of Medical Sciences, Yazd, Iran; ^2^Department of Nutrition, Faculty of Health, Shahid Sadoughi University of Medical Sciences, Yazd, Iran; ^3^Center for Healthcare Data Modeling, Department of Biostatistics and Epidemiology, School of Public Health, Shahid Sadoughi University of Medical Sciences, Yazd, Iran; ^4^Yazd Cardiovascular Research Center, Non-communicable Disease Research Institute, Shahid Sadoughi University of Medical Sciences, Yazd, Iran; ^5^Industrial Diseases Research Center, Shahid Sadoughi University of Medical Sciences, Yazd, Iran

**Keywords:** hypertension, dietary pattern, dietary analysis methods, principal component analysis, partial least-squares, reduced-rank regressions

## Abstract

Limited data exist on the advantage of data reduction hybrid methodologies for evaluating the relationship between dietary patterns (DPs) and chronic diseases and they have led to inconsistent results. This study aimed to investigate the association between DPs extracted using principal component analysis (PCA), partial least-squares (PLS), and reduced-rank regressions (RRRs) in identifying DPs associated with hypertension (HTN) risk. The current study was conducted in the context of two cohort studies accomplished in Iran. DPs were generated using PCA, PLS, and RRR methods. Log-binomial logistic regression test was used to assess the association between DPs and the risk of HTN. From a total of 12,403 included participants aged 20–70 years, 507 incident cases of confirmed HTN were identified. The PCA-DP2 was associated with HTN in the fully adjusted model (T3 vs. T1: RR: 0.737, 95% CI: 0.57–0.93, *P*_trend_ = 0.013). The PLS-DP2 and HTN risk were inversely associated in the multivariate model (T3 vs. T1: RR: 0.704, 95% CI: 0.54–0.91, *P*_trend_ = 0.013). The RRR-DP2 was associated with an increased risk of HTN (T3 vs. T1: RR: 1.412, 95% CI: 1.11–1.80, *P*_trend_ = 0.007). Our findings suggest that the RRR method reveals stronger results in association with HTN risk. However, further investigations are required to confirm the association between DPs derived by PLS and RRR methods by incorporating biomarkers related to HTN as the response variables.

## Introduction

Hypertension (HTN), or elevated blood pressure, is a leading risk factor for cardiovascular diseases (CVDs) and premature death (1). It is estimated that 1.28 billion adults aged 30–79 years have HTN, and its prevalence is increasing worldwide (2). Evidence has well established that HTN is associated with genetic and environmental factors such as obesity, tobacco use, sedentary lifestyle, and diet (3). A healthful lifestyle is a key strategy for the prevention of HTN, and diet is known as a modifiable lifestyle component with the strongest impact on blood pressure (4).

The majority of previous studies that have investigated the relationship between diet and HTN focused on the consumption of a single nutrient, food item, or food group (5, 6); however, more recently, nutritional epidemiologic investigations of chronic diseases have gone beyond these approaches, focusing on evaluating the effect of the overall diet (7).

A dietary patterns (DPs) approach, as a complementary approach, can provide helpful findings, translating into dietary guidelines without fully understanding the mechanisms underlying the observed relationships (7). Most previous studies have investigated DPs generated by principal component analysis (PCA) (8). Recently, two alternative reduction techniques have been proposed for DPs research: reduced rank regression (RRRs) and partial least-square analysis (PLS) (9). The RRR creates linear combinations of predictor variables with as much variation as possible in a combined set of response variables including biomarkers or macro/micronutrients (9). The PLS is a compromise between the PCA and RRR methods which explain variation in predictor variables and selected intermediate response variables (9). The main advantage of these methods is that the derived DPs are correlated with biological pathways related to the etiology of diseases (9).

Few studies have assessed the relationship between DPs and HTN *via* the RRR method (10, 11). In a cross-sectional study by da Silva et al. (10) was found that healthier DP from the RRR method was directly related to HTN in women 40–60 years. There is no previous study investigating HTN development risk and the PLS method, specifically. However, Naja et al. (12) reported that the western and traditional Lebanese DPs were associated with higher HTN risk in Lebanese men. Previous studies suggested that using the RRR method resulted in a more significant link with the HTN compared to PCA and PLS methods (13, 14), whereas, PCA-derived patterns were more associated with the real dietary habits in their study population (9).

Therefore, the main objective of the present study was to assess whether two hybrid methods with intermediate variables for HTN generate different DPs (RRR and PLS) compared to a common method (PCA).

## Materials and methods

### Study design and study population

The Yazd Health Study (YaHS) was conducted in September 2014 in Yazd greater area located in central Iran, including 9,962 adults aged 20–70 years were participated in the enrollment phase. The assessment of dietary intake was separately collected in Taghzieh Mardom Yazd (TAMYZ) study by using a validated semi-quantitative food frequency questionnaire (FFQ) (15). The Shahedieh cohort study is a part of a large Persian multicentral cohort study (Persian cohort) performed on 180,000 participants in 18 various geographical areas of Iran (16). The Shahedieh study was established in 2014 and 9,977 adults aged 35–70 years were enrolled to the study at baseline. To collect the dietary intake of participants, a semi-quantitative FFQ was completed. Data on demographic characteristics, medical history, smoking status, and physical activity was collected in both cohort studies. The study protocol for YaHS-TAMYZ (17) and Shahedieh (16) studies are completely described elsewhere.

The exclusion criteria were: participants who reported incomplete dietary intakes data or an implausible total energy intake < 800 kcal/d or ≥ 6,000 kcal/d (YaHS-TAMYZ study, *n* = 1,692, Shahedieh study, *n* = 1,708), those who had a previous diagnosis of HTN (YaHS-TAMYZ study, *n* = 1,773, Shahedieh study, *n* = 2,054), those who had not provided data on National Identifier (YaHS-TAMYZ study, *n* = 287, Shahedieh study, *n* = 0), had no follow-up data (YaHS-TAMYZ study, *n* = 0, Shahedieh study, *n* = 0), and people who died (YaHS-TAMYZ study, *n* = 50, Shahedieh study, *n* = 28) were excluded, which left 12,403 participants (YaHS-TAMYZ study: 6,216, Shahedieh study: 6,187) for current analyses.

All participants filled an informed consent before enrolling the cohort studies. Both studies were approved by the research Council of Shahid Sadoughi University of Medical Sciences. The present study was ethically approved by Shahid Sadoughi University’s Ethics Committee (approval code: IR.SSU.SPH.REC.1399.197).

### Dietary assessment and food groups

Dietary intake in the YaHS-TAMYZ study was examined by a validated, multiple-choice semi-quantitative FFQ (15). The food item list consisted of 178 food or food groups representing commonly consumed foods in Yazd. Participants were asked by trained interviewers to report the frequency of food item intake during the past year by answering 10-multiple-choice frequency responses ranging from “never or less than once a month” to “10 or more times per day.” Moreover, the FFQ had five choices for portion size for estimating the intake of each food item (18). In Shahedieh study, dietary intake information was collected by a semi-quantitative open-ended FFQ based on 134-items through face-to-face interviews with the participants. Participants of the Shahedieh study were asked to report how often on average over the past year they consumed a typical portion size of each food item with multiple possible responses on a “daily,” “weekly,” or “monthly” basis. The frequency and portion size reported for each food item were converted to grams per day using household measures (19). The United States Department of Agriculture (USDA) food composition database was used to estimate daily intake of energy and nutrient for each participant (20). Food items were merged into 32 food groups based on food items similarity in their nutrient profiles and are presented in Table 1.

**TABLE 1:** Food grouping used in the dietary pattern (DP) analyses in the Yazd Health Study-Taghzieh Mardom Yazd (YaHS-TAMYZ) and Shahedieh cohort studies.

Food groups	Food items
Processed meats	Sausages
Red meats	Beef, hamburger, lamb
Organ meats	Beef liver and lamb organ (tongue, tripe, head and trotters, brain, foot, abomasum)
Fish	Canned tuna fish, other fish
Poultry	Chicken with skin, chicken without skin, chicken with or without skin (liver, heart, gizzard)
Eggs	Eggs
Margarine	Margarine
Dairy products	Milk, yogurt, cheese, curd, ice-cream, flavored milk, chocolate milk, coffee milk, honey milk, cream, butter
Tea	Tea
Coffee	Coffee
Fruits	Pears, apricots, cherries, apples, raisins or grapes, bananas, cantaloupe, watermelon, oranges, grapefruit, kiwi, grapefruits, strawberries, peaches, nectarine, tangerine, mulberry, plums, persimmons, pomegranates, lemons, pineapples, fresh figs, and dates
Fruit juice	Apple juice, orange juice, grapefruit juice, other fruit juices
Tomatoes	Tomatoes, tomato pasta
Other vegetables	Cucumber, cabbage, cauliflower, Brussels sprouts, kale, carrots (row or boiled), squash, spinach, lettuce, mixed vegetables (row or cooked), eggplant, celery, kohlrabi, green peas, green beans, turnip, corn, mushrooms, onions, beet, beet root, artichokes, bell pepper, pepper
Garlic	Garlic
Potatoes	Potatoes, French fries
Whole grains	Iranian dark bread (sangak, taftoon, barbari), local bread (korno, tanoori), wheat germ, oatmeal, barley, bulgur, whole grain biscuit (saghe talaee)
Refined grains	White breads (Lavash, Baguettes), noodles, pasta, rice, biscuits, and wafers
Pizza	Pizza
Snacks	Potato chips, puffs, crackers, popcorn
Dried fruit	Dried figs, dried dates, dried mulberries, and other dried fruits
Mayonnaise	Mayonnaise
Nuts	Peanuts, almonds, pistachios, hazelnuts, walnuts, sunflower, pumpkin, and watermelon seeds
Olive	Olives and olive oils
Sweet dessert	Chocolates, cookies, cakes, confections, traditional sweets (kamache sen, poshtzik, pirashki, qottab, baqlava, loz, haji badam, nan berenj, sohan, yazdi cake), ardeh (liquid sesame), homemade halva, halva shekari (a sweet breakfast food in Iran), creme caramel, homemade cake
Hydrogenated fats	Hydrogenated fats, animal fats
Vegetables oils	Vegetables oils (except for olive oil)
Sugars	Jam, honey, sugars, candies, syrup, Nabat (an Iranian confectionery made of sugar and served by tea), noql (an Iranian confectionery), pashmak
Soft drinks	Soft drinks, non-alcoholic beer, all types of artificial fruit juices
Yogurt drink	Yogurt drink
Pickles	Pickles
Legumes	Beans, peas, lima beans, broad beans, lentils, soy, split peas, mung beans

### Assessment of other covariates

To collect data on age, sex, educational status, marital status, smoking status, and the history of chronic diseases, a similar demographic questionnaire in both YaHS-TAMYZ and Shahedieh cohort studies was used.

Five categories (20–30, 30–40, 40–50, 50–60, and ≥ 60 years) was considered for age variable. Educational status of participants was classified into four levels (Uneducated, Elementary or guidance school, High school diploma, and B.Sc. or higher academic degree). Smoker participants were defined as current smokers, while non-smoker participants categorized as former smokers and never smokers. Marital status was defined into three categories (Single, Married, and Widowed or divorced).

The height and body weight of the study participants were measured in both YaHS-TAMYS and Shahedieh studies. In the YaHS-TAMYS study, body weight (kg) was measured by using an Omron BF511 portable digital scale (Omron Inc., Nagoya, Japan) with the accuracy of 0.1 kg, while standing on the middle of the scale, without assistance and with minimum clothing and height (cm) was measured in a standing position using a tape measure on a straight wall to the nearest centimeter. In the Shahedieh study, body weight (kg) and height (cm) were measured by using the National Institute of Health protocols by trained staffs. Body weight was measured while the participants were with minimum clothing and without shoes by using a digital scale (SECA, model 755, Germany). Height was measured by using a measure tape attached to a flat wall with the accuracy of 0.5 cm. Body mass index (BMI) was calculated as weight (kg)/height squared (m^2^).

In the YaHS-TAMYZ cohort study, the short version of the International Physical Activity Questionnaire (IPAQ) was used to measure physical activity level (21). In the Shahedieh cohort study, participants were asked about their usual physical activity levels in the last year and in case they had seasonal jobs (22). Physical activity was expressed as metabolic equivalent hours per week (MET-h/wk).

### Clinical assessment

#### Follow-up of study participants and case confirmation

In the YaHS-TAMYZ cohort study, information on death events was collected using data from population-based registries and linked outcome information from the aggregated hospital information system (Samanah Electronici PArvandeh Salamat-SEPAS) which covers 100% of public hospitals and the majority of private hospitals in Yazd province. The data was obtained from SEPAS by using the National Identifier number of each participant to link data.

During follow-up time in the Shahedieh cohort study, participants received annual phone calls and follow-up questionnaires were completed in terms of the death events. Moreover, a verbal autopsy form validated in the Iranian population was completed during the death events. Two trained internists examined the medical documents to determine the cause of death. In case of inconsistency, a third internist performed a final assessment of the documents to reach a final decision.

In both studies, new cases of HTN were defined as individuals reporting a physician-based diagnosis of HTN and antihypertensive medication use in the annual follow-up phone call who did not have HTN at baseline.

### Statistical analysis

#### Dietary patterns analysis

To identify DPs out of 32 food groups, three complementary data reduction techniques, including PCA, PLS, and RRR were used. An explained variance calculates the proportion of variation in a DP which can be attributable to the food groups or responses. In the PCA method, the DPs explain as much variation as possible of the food groups. To obtain the variance of the response variables explained by the DPs derived from the PCA method, linear regression analysis of the DPs scores, and response variables was used. The RRR method identifies linear functions of predictors (food groups) which explain as much intermediate responses variation as possible using a covariance matrix of predictors and responses in calculating the DPs scores. The PLS method combines PCA and RRR methods and calculates DPs scores considering both the predictor and response matrices; therefore, the explained variance of both food groups and response variables is expected to be between the PCA and RRR methods.

The number of DPs initially generated by PCA is constrained by the number of food groups used (23); nevertheless, we retained only three DPs from PCA for subsequent analyses based on the scree plot, an eigenvalue (>1), and the interpretability of the principal DPs (24). To obtain orthogonal DPs and increase the interpretability of principle DPs, Varimax rotation was applied. Kaiser–Meyer–Olkin (KMO) test was performed for checking sample adequacy.

Based on previous studies, dietary intake of sodium, potassium, and saturated fatty acids were selected as the response variables for PLS and RRR.

The SAS procedure PLS was used to conduct PLS and RRR analysis. The number of DPs derived by PLS and RRR is restricted by the number of intermediate response variables used; thus, three DPs were specified in each method. For both PLS and RRR, we calculated the continuous DPs scores (the linear functions of food groups) in the subsequent analyses and interpretations.

#### Descriptive analyses and modeling

Quantitative and qualitative variables were compared between participants with and without HTN by using independent sample *t*-test and chi-square tests, respectively. For further analyses, DP scores were categorized into tertiles. To assess the association between DPs derived by PCA, PLS, and RRR analyses and the risk of HTN incidence, binomial logistic regression was used. In all analyses, in addition to the crude model, the first model was adjusted for age, sex, and energy intake; the second model was additionally controlled for education, marital status, smoking status, and physical activity; and in the third model, BMI was additionally adjusted.

All statistical analyses were performed with SAS Version 8.02 (SAS Institute, Cary, NC, USA) software. *P* values less than 0.05 were considered statistically significant.

#### Comparison of dietary pattern methods

A comparison between PCA, PLS, and RRR methods was conducted according to the relative factor loading within each DPs and its association with HTN risk. In addition, these three methods were examined based on the magnitude of variation of each method which explained the food groups and responses.

## Results

Of the 12,403 participants included in the study analyses, about 54% were men (Table 2). After 6 years of follow-up for the YaHS-TAMYZ study and 4 years for the Shahedieh study, 507 participants were diagnosed with HTN. In addition, Table 2 represents the baseline characteristics of the study population. In addition, age, sex, and energy-adjusted dietary macronutrients and micronutrients intake of the participants according to tertiles of DPs derived by three methods are presented in the Supplementary Tables 1–3.

**TABLE 2:** Baseline characteristics of participants with and without hypertension (HTN)^a,b^.

	Participants with HTN (*n* = 507)	Participants without HTN (*n* = 11,896)	*P*-value
**Age category [*n* (%)]**			<0.001
20–29 (years)	7 (1.4)	1,522 (12.8)	
30–39	46 (9.1)	3,281 (27.6)	
40–49	151 (29.7)	3,562 (29.9)	
50–59	184 (36.4)	2,295 (19.3)	
≥60	119 (9.3)	1,236 (10.5)	
**Sex [*n* (%)]**			<0.001
Male	208 (41)	5,989 (50.3)	
Female	299 (59)	5,907 (49.7)	
**Marital status [*n* (%)]**			<0.001
Single	5 (1)	851 (7.2)	
Married	477 (94.1)	10,728 (90.2)	
Widow or divorced	25 (4.9)	317 (2.7)	
**Educational status [*n* (%)]**			<0.001
Uneducated	133 (26.2)	1,917 (16.1)	
Elementary or guidance school	228 (45)	4,562 (38.3)	
High school diploma	92 (18.1)	3,313 (27.8)	
B.Sc. or higher academic degree	54 (10.7)	2,104 (17.7)	
**Smoking status [*n* (%)]**			0.041
Current smoker	46 (9.1)	1,471 (12.4)	
Former smoker	28 (5.5)	506 (4.3)	
Never smoker	433 (85.4)	9,919 (83.4)	
**Body mass index (kg/m^2^)**	29.48 ± 4.76	27.16 ± 4.95	<0.001
**Physical activity (MET-h/wk^c^)**	47.75 (38.33, 731.81)^d^	46.9 (37.70, 519.75)^d^	0.113
**Total energy intake (kcal)**	2,960.52 ± 1,242.57	3,059.35 ± 1,207.62	0.071

^a^Values are mean ± standard deviation (SD) or percentage (%).

HTN, hypertension; MET, metabolic equivalent.

^b^*P*-values are resulted from independent *t*-test for quantitative variables and from chi-square for qualitative variables.

^c^Mann–Whitney test was used.

^d^Values are median interquartile range (IQR).

### Dietary patterns derived by PCA, PLS, and RRR methods

Dietary patterns factor loadings of the 32 food groups for the PCA, PLS, and RRR are presented in Table 3. Using the PCA method, three DPs were generated characterized by: PCA-DP1: high intake of processed meats, organ meats, fish, margarine, fruit juice, pizza, snacks, sweet dessert, and soft drinks, and low intake of whole grains; PCA-DP2: high intake of red meats, dairy products, fruits, tomatoes, potatoes, refined grains, vegetable oils, and pickles; PCA-DP3: high intake of tea, nuts, mayonnaise, snacks, sweet dessert, soft drinks, and sugars.

**TABLE 3:** Factor loadings of food groups in dietary patterns identified using principal component analysis (PCA), partial least-squares (PLS), and reduced-rank regression (RRR) methods.

Food groups	PCA			PLS			RRR		
	DP 1	DP 2	DP 3	DP 1	DP 2	DP 3	DP 1	DP 2	DP 3
Processed meats	–	–	–	–	–	–	–	0.261	–
Red meats	–	–	–		–	–	–	–	–
Organ meats	0.285	–	–	–	–	–	–	–	–
Fish	–	–	–	–	–	–		–	–
Poultry	–	–	–	–	–	–		–	–
Eggs	–	–	–	–	–	–	–	–	–
Margarine	–	–	–	–	–	–	–	0.343	−0.260
Dairy products	–	0.250	–	0.281	–	–	0.357	–	−0.292
Tea	–	–	0.359	–	0.252	–	–	–	–
Coffee	–	–	–	–	–	–	–	–	–
Fruits	–	0.336	–	0.288	–	−0.401	0.321	−0.455	−0.278
Fruit juice	–	–	–	–	–	−0.332	–	–	−0.279
Tomatoes	–	0.326	–	–	–	–	–	–	–
Other vegetables	–	–	–	0.319	–	–	0.329	–	–
Garlic	–	–	–	–	–	–	–	–	–
Potatoes	–	0.278	–	–	0.268	–	–	–	–
Whole grains	−0.258	–	–	–	0.382	–	–	–	0.419
Refined grains	–	–	–	–	0.296	–	–	–	–
Pizza	–	–	–	–	–	–	−0.249	–	–
Snacks	0.269	–	–	–	–	–	–	–	–
Dried fruit	–	–	–	–	–	−0.332	–	–	–
Mayonnaise	–	–	0.256	–	–	–	–	–	–
Nuts	–	–	0.334	–	–	–	–	–	–
Olive	–	–	–	–	–	–	–	–	–
Sweet dessert	0.382	–	–	0.278	−0.314	–	–	0.278	–
Hydrogenated fats	–	–	–	–	–	0.253	–	–	–
Vegetables oils	–	–	–	–	–	–	–	–	–
Sugars	–	–	0.498	–	–	0.255	–	–	0.273
Soft drinks	–	–	0.347	–	–	–	–	–	–
Yogurt drink	–	–	–	–	–	–	–	–	–
Pickles	–	–	–	–	–	–	–	–	–
Legumes	–	–	–	–	–	–	–	–	–

Factor loadings < |0.25| were deleted for simplicity. DP, dietary pattern.

The percentage of explained variance by the DPs is presented in Table 4. The three PCA-derived DPs accounted for 11.165, 7.024, and 5.251%, respectively of the variance in food groups and 10.524, 25.338, and 4.086% of the variance in the response variables, respectively.

**TABLE 4:** Explained variation in food groups and responses using principal component analysis (PCA), partial least-squares (PLS), and reduced-rank regression (RRR).

	Explained variation in food groups			Explained variation in responses		
	PCA	PLS	RRR*	PCA	PLS	RRR**
**DP 1**	11.165	8.818	6.798	10.524	39.863	46.840
**DP 2**	7.024	8.440	4.074	25.338	6.645	9.147
**DP 3**	5.251	3.805	4.339	4.086	7.324	2.718
Total	23.442	21.064	15.212	39.949	53.832	58.705

*Food items from the food frequency questionnaire (FFQ) were defined as 32 food groups (Table 1).

**Response variables were sodium intake, potassium intake, and saturated fatty acid intake.

The first PLS DP, labeled “PLS-DP1,” was characterized by a high intake of organ meats, dairy products, fruits, fruit juice, other vegetables, dried fruit, nuts, and sweet dessert. A high intake of tea, tomatoes, potatoes, refined grains, whole grains, and vegetable oils and a low intake of sweet dessert, processed meats, organ meats, and margarine characterized the second PLS DP, “PLS-DP2.” The third PLS DP, identified as “PLS-DP3” had high intakes of refined grains, whole grains, hydrogenated fats, and sugars and a low intake of fruits, fruit juice, other vegetables, and dried fruit. The three PLS DPs explained 39.863, 6.645, and 7.324% of the variance in the response variables, respectively.

Regarding PLS results, all DPs accounted for 21.064% of the variation in food groups (8.818, 8.440, and 3.805%, respectively).

The first DP derived by the RRR method (RRR-DP1) was characterized by high intakes of dairy products, fruits, other vegetables, mayonnaise, sweet dessert, and low intake of pizza, and snacks explained 46.840% of the variation in the response variables. The second RRR-derived DP labeled “RRR-DP2” was characterized by a high intake of processed meats, red meats, margarine, hydrogenated fats, and sweet dessert, and a low intake of fruits, tomatoes, other vegetables, whole grains, and mayonnaise, which explained 9.147% of the variance in the responses. The third DP (RRR-DP3) was characterized by a high intake of whole grains, nuts, and sugars and a low intake of red meats, organ meats, margarine, dairy products, fruits, and fruit juice, which explained 2.718% of the variance in the responses.

Findings from the RRR method indicated that three DPs explained 15.212% of the total variation in food groups (6.798, 4.074, and 4.339%, respectively).

### Association between dietary patterns and HTN

Table 5 represents the risk of HTN for each tertile of the DP scores. Highest adherence to the PCA-DP1 and the PCA-DP3 in comparison with lowest adherence were associated with a decreased risk of HTN only in the crude model (RR: 0.788, 95% CI: 0.63–0.98, *P*_trend_ = 0.013 and RR: 0.728, 95% CI: 0.57–0.93, *P*_trend_ = 0.013, respectively). PCA-DP2 was significantly associated with HTN in the crude and all adjusted models (crude model_T3vs_._T1_: RR = 0.733, 95% CI: 0.57–0.94, *P*_trend_ = 0.011; Model I_T3vs_._T1_: RR = 0.770, 95% CI: 0.60–0.98, = 0.034; Model II_T3vs_._T1_: RR = 0.756, 95% CI: 0.59–0.96, *P*_trend_ = 0.024, and Model III_T3vs_._T1_: RR = 0.737, 95% CI: 0.57–0.93, *P*_trend_ = 0.013).

**TABLE 5:** Risk ratios and 95% confidence intervals for hypertension (HTN) and dietary patterns (DPs) derived using principal component analysis (PCA), partial least-squares (PLS), and reduced-rank regression (RRR).

	Tertile1	Tertile2	Tertile3	*P* trend
**PCA**	*n* = 4,124	*n* = 4,137	*n* = 4,142	
**Dietary pattern 1**				
Crude	1	0.858 (0.69, 1.06)	**0.788 (0.63, 0.98)**	0.013
Model I	1	1.065 (0.86, 1.32)	1.129 (0.90, 1.41)	0.510
Model II	1	1.083 (0.87, 1.35)	1.166 (0.91, 1.48)	0.369
Model III	1	1.057 (0.84, 1.31)	1.160 (0.91, 1.47)	0.411
**Dietary pattern 2**				
Crude	1	0.875 (0.71, 1.07)	**0.733 (0.57, 0.94)**	0.011
Model I	1	0.924 (0.75, 1.13)	**0.770 (0.60, 0.98)**	0.034
Model II	1	0.914 (0.74, 1.12)	**0.756 (0.59, 0.96)**	0.024
Model III		0.905 (0.73, 1.11)	**0.737 (0.57, 0.93)**	0.013
**Dietary pattern 3**				
Crude	1	0.987 (0.80, 1.22)	**0.728 (0.57, 0.93)**	0.013
Model I	1	1.093 (0.88, 1.34)	0.858 (0.67, 1.09)	0.256
Model II	1	1.090 (0.88, 1.34)	0.862 (0.67, 1.10)	0.270
Model III	1	1.109 (0.90, 1.36)	0.884 (0.69, 1.12)	0.383
**PLS**	*n* = 4,130	*n* = 4,134	*n* = 4,139	
**Dietary pattern 1**				
Crude	1	0.903 (0.73, 1.11)	**0.689 (0.52, 0.90)**	0.006
Model I	1	1.069 (0.87, 1.31)	0.898 (0.68, 1.17)	0.496
Model II	1	1.070 (0.87, 1.31)	0.904 (0.69, 1.18)	0.527
Model III	1	1.067 (0.87,1.31)	0.879 (0.67, 1.14)	0.379
**Dietary pattern 2**				
Crude	1	**0.737 (0.59, 0.92)**	1.037 (0.81, 1.32)	0.734
Model I	1	**0.646 (0.51, 0.80)**	**0.784 (0.61, 0.99)**	0.056
Model II	1	**0.625 (0.49, 0.78)**	**0.738 (0.56, 0.95)**	0.031
Model III	1	**0.622 (0.49, 0.78)**	**0.704 (0.54, 0.91)**	0.013
**Dietary pattern 3**				
Crude	1	1.043 (0.84, 1.29)	**0.734 (0.57, 0.95)**	0.029
Model I	1	1.185 (0.96, 1.46)	0.938 (0.73, 1.20)	0.746
Model II	1	1.176 (0.95, 1.45)	0.937 (0.73, 1.20)	0.740
Model III	1	1.223 (0.99, 1.51)	0.977 (0.76, 1.25)	0.976
**RRR**	*n* = 4,134	*n* = 4,131	*n* = 4,138	
**Dietary pattern 1**				
Crude	1	0.829 (0.66, 1.03)	**0.692 (0.52, 0.92)**	0.019
Model I	1	0.972 (0.78, 1.20)	0.875 (0.65, 1.16)	0.463
Model II	1	0.967 (0.77, 1.20)	0.876 (0.65, 1.16)	0.464
Model III	1	0.971 (0.78, 1.20)	0.856 (0.64, 1.14)	0.373
**Dietary pattern 2**				
Crude	1	0.805 (0.64, 1.00)	0.929 (0.73, 1.17)	0.545
Model I	1	1.016 (0.81, 1.27)	**1.299 (1.03, 1.64)**	0.033
Model II	1	1.036 (0.82, 1.29)	**1.348 (1.05, 1.72)**	0.020
Model III	1	1.068 (0.85, 1.33)	**1.412 (1.11, 1.80)**	0.007
**Dietary pattern 3**				
Crude	1	1.052 (0.84, 1.31)	1.016 (0.78, 1.32)	0.993
Model I	1	1.056 (0.84, 1.32)	1.032 (0.79, 1.33)	0.836
Model II	1	1.038 (0.82, 1.29)	1.018 (0.78, 1.32)	0.911
Model III	1	1.061 (0.85, 1.32)	1.054 (0.81, 1.37)	0.691

Crude: adjusted for energy intake. Model I: additionally, adjusted for age and sex. Model II: additionally, adjusted for physical activity, smoking status, educational status, marital status. Model III: additionally, adjusted for body mass index (BMI). Bolded values show significant associations.

The RRs for the HTN in the crude model was 0.689 (95% CI: 0.52–0.90, *P*_trend_ = 0.006) and 0.734 (95% CI: 0.57–0.95, *P*_trend_ = 0.029) for the participants in the highest tertile compared to those in the lowest tertile of adherence to the PLS-DP1 and the PLS-DP3, respectively; however, these associations were not observed in all adjusted models. The association the PLS-DP2 was inversely significant in all adjusted models with HTN risk (Model I_T3vs_._T1_: RR = 0.784, 95% CI: 0.61–0.99, *P*_trend_ = 0.056; Model II_T3vs_._T1_: RR = 0.738, 95% CI: 0.56–0.95, *P*_trend_ = 0.031, and Model III_T3vs_._T1_: RR = 0.704, 95% CI: 0.54–0.91, *P*_trend_ = 0.013).

Compared with the participants in the lowest tertile, those in the third tertile of RRR-DP2 adherence had 1.412 times (95% CI: 1.11–1.80, *P*_trend_ = 0.007) higher risks of HTN after adjustment for all confounders. In addition, this association was also significant in Model I_T3vs_._T1_ (RR = 1.299, 95% CI: 1.03–1.64, *P*_trend_ = 0.033) and Model II_T3vs_._T1_ (RR = 1.348, 95% CI: 1.05–1.72, *P*_trend_ = 0.020). Highest adherence to RRR-DP1 in comparison to lowest adherence was associated with a reduced risk of HTN only in crude model_T3vs_._T1_ (RR = 0.692, 95% CI: 0.52–0.92, *P*_trend_ = 0.019).

## Discussion

The aim of our study was to investigate the potential association between DPs and the risk of HTN incidence using three different methods to generate DPs. The PCA-DP2 and the PLS-DP2 were significantly associated with HTN, however, compared to the participants in the lowest tertile, those in the third tertile of the RRR-DP2 adherence had 1.414 times higher odds of HTN incidence. Though all three DPs have an association with HTN risk, the RRR method showed a stronger statistical relationship.

Our findings showed that the highest adherence to the RRR-DP2 in comparison to the lowest adherence was associated with an increased risk of HTN. The RRR-DP2 was characterized by high intakes of processed and red meats, margarine, hydrogenated fats, and sweet dessert and a low intake of fruits, tomatoes, other vegetables, whole grains, and mayonnaise. In line with our findings, Du and Cao (25) reported that RRR derived-DP associated with C-reactive protein (CRP), which was high in red meat, snacks, and nuts, and low in grains, poultry, and fish led to a higher risk of high-normal and high levels of systolic blood pressure. In contrast, da Silva et al. (10) found that the RRR-derived DP associated with sodium, potassium, and saturated fat is characterized by a high intake of healthy food items such as banana, papaya, apple, orange, pumpkin, green leafy vegetables, other vegetables, and natural fruit juice in contrast with a low intake of unhealthy foods such as processed meat presented a direct association with HTN women between 40 and 60 years.

The mechanisms of red meat intake in increasing blood pressure are complex and attributed to multiple factors. The high content of saturated fatty acids and cholesterol in red meat has been directly associated with HTN in previous studies (26, 27). It is demonstrated that dietary cholesterol is directly associated with endothelial dysfunction and arterial stiffness (28). Evidence from animal studies has shown that increased oxidative stress in cholesterol-fed rabbits was strongly attributed to endothelial dysfunction (29). In addition, some evidence has demonstrated that Maillard reaction products during cooking or processing of meat, such as advanced glycosylation end-products and heterocyclic amines, may induce mediators of inflammation and oxidation pathways and impair insulin activity which can contribute to elevated blood pressure (23, 30). A direct association between dietary advanced glycosylation endproducts (AGEs) and CRP, tumor necrosis factor-a (TNF-a), vascular cell adhesion molecule 1, and 8-isoprostane (24, 31) is proposed. Vascular damage caused by AGEs can lead to increased vascular and myocardial stiffening, oxidative stress, and inflammation (32). Moreover, HTN is related to the low circulating level of the soluble receptor for AGE (23). The metabolism of L-carnitine, as abundant trimethylamine in red meat, by gut microbiota produces trimethylamine-N-oxide and led to atherosclerosis in mice (33). Though, processed meats are rich in sodium and nitrite contents, which are used as food preservatives (34); it is established that sodium intake has the main effect on increasing blood pressure (35).

Several prospective cohort studies have shown that long-term consumption of all types of fruits was relative to a reduced risk of HTN in adults. Tsubota-Utsugi et al. (36) found that subjects in the highest quintile in comparison to those in the lowest quintile of fruit consumption had 60% lower odds of HTN. There are several mechanisms of fruits and vegetables related to reducing blood pressure. The high content of polyphenols like flavonoids and anthocyanins in some fruits and vegetables can reduce the risk of HTN (37, 38). In addition, it has been revealed that grape polyphenols may contribute to vasorelaxation and decrease blood pressure (39).

The findings of a meta-analysis by Schwingshackl et al. (40) showed a significant inverse relationship between whole grain consumption and HTN in the linear and non-linear dose-response analysis. Sawicki et al. (41) reported that whole grain intake was inversely associated with systolic blood pressure, but not refined grains intake. In addition, a meta-analysis of prospective studies revealed an inverse association between whole grain intake and HTN risk, although no significant link was observed between refined grains intake and the risk of HTN (40). According to the previous evidence, the components of whole grains, including phytochemicals, fiber, and minerals like potassium, magnesium, zinc, and selenium can decrease blood pressure (42). Moreover, the high content of fiber in whole grains can prevent the development of HTN through the alteration in gut microbiota (43).

In the present study, high dairy product intake is one of the features of the second PLS-derived DP associated with a lower risk of HTN. This finding supports the previous evidence on the inverse relationship between dairy intake and HTN risk. Several mechanisms may involve in contributing to the beneficial effect of dairy products on blood pressure. Dairies are rich in calcium, magnesium, potassium, and phosphorus and all of them are contributed to reduce blood pressure through vascular resistance regulation and promotion of vasodilation (44). Potential evidence proposed that bioactive peptides from dairy protein may inhibit the effect of angiotensin I–converting enzyme on constriction of blood vessels (45).

In the present study, the PLS-DP2 which was high in tea, tomatoes, potatoes, refined grains, whole grains, and vegetable oils, but low in processed meats, organ meats, margarine, and sweet dessert was inversely related to a lower risk of HTN. The food groups loaded in this DP are similar to the RRR-DP2, and both of them are associated with our selected response variables including sodium, potassium, and saturated fatty acids. One of the specific food items of the PLS-DP2 related to blood pressure is tea consumption. Evidence has shown that habitual tea drinkers had a 14% lower risk of HTN incidence in comparison with non-habitual tea drinkers (46). It is demonstrated that tea had anti-inflammatory and endothelial function improvement effects through a wide range of antioxidants and polyphenols like flavonoids (47).

The DP derived by the PCA method (PCA-DP2) associated with a lower risk of HTN was a mix of healthy and unhealthy food groups of usual DPs, including a high intake of red meats, dairy products, fruits, and tomatoes, potatoes, refined grains, vegetable oils, and pickles. In a similar way, in a cross-sectional study of India, the “fruits-vegetables-sweets-snacks” pattern had a positive link with diastolic blood pressure (48). Given the interpretation of the DPs, the PCA-derived DPs reflected a wide extent of the dietary behavior of the studied population (9). The behavior-related DPs derived by the PCA method cannot predict the risk of diseases (9).

There are some previous studies that have found that RRR-derived DPs had a stronger link with the disease compared to those derived by PCA or PLS; it clearly could be explained because DPs from PLS and RRR methods are generated *via* disease-related response variables. Naja et al. (12) reported that a western DP from all PCA, PLS, and RRR methods was associated with an increased risk of HTN in the Lebanese population. However, a cross-sectional study by DiBello et al. (49) showed that PCA generated more myocardial infarction–associated DPs than RRR that used nutrient intake as response variables.

There are several limitations to the present study. In our study, the dietary intakes were evaluated using the FFQ which is prone to measurement error. Moreover, data on dietary intake was collected only at the baseline of both cohort studies and we could not warrant that participants have not changed their diets during the follow-up time. The follow-up time was 6 years for the YaHS-TAMYZ study and 4 years for the Shahedieh study; although the follow-up time is considered in the analyses, its difference between the two studies might be considered as a limitation. Moreover, both cohort studies had less than a 6-year follow-up time; hence, we might not reveal the long-term effects of DPs on HTN risk. Additionally, the limited number of incident HTN cases could affect the observed associations in the present study. In addition, using different evaluation instruments in the two cohorts could cause bias in the analyses. Furthermore, the IPAQ is only validated on individuals up to 65 years of age, however, the age range of the present study is higher and physical activity data collected on participants over 65 are not valid. We tried to adjust a wide variety of confounders, however, the role of residual confounding factors cannot be completely ruled out in observational studies.

Altogether, DP derived by PCA associated with HTN risk was more similar to common dietary behavior of the studied population and DPs derived by PLS and RRR methods reflected food groups in association with response variables. Though all three DPs have an association with HTN risk, the RRR method showed a stronger statistical relationship. However, further investigations are required to confirm the association between DPs derived by the PLS and the RRR methods by incorporating biomarkers related to HTN as the response variables.

## Data availability statement

The raw data supporting the conclusions of this article will be made available by the authors, without undue reservation.

## Ethics statement

The studies involving human participants were reviewed and approved by the Shahid Sadoughi University of Medical Sciences. The patients/participants provided their written informed consent to participate in this study.

## Author contributions

AS-A and SB conceived and designed the study. SJ, SB, and AS-A conducted and interpreted the statistical analyses. SB wrote the first draft of the manuscript. AS-A, SJ, SK, MM, and AM critically reviewed the manuscript. All authors read and approved the final version of the manuscript.
