# Supplementary material for: A comparison of principal component analysis, partial least-squares, and reduced-rank regressions in the identification of dietary patterns associated with hypertension: YaHS-TAMYZ and Shahedieh cohort studies
Source: Front Nutr. 2023 Jan 12;9:1076723. doi: 10.3389/fnut.2022.1076723 (PMC9879482; doi:10.3389/fnut.2022.1076723)
Supplement: Supplementary file 1 [file Table_6.DOCX]

| **Supplementary table 1.** Age, sex, and energy-adjusted dietary macronutrients and micronutrients intake of study participants according to tertiles of dietary patterns derived by PCA. | | | | | | | | | | | | |
| --- | --- | --- | --- | --- | --- | --- | --- | --- | --- | --- | --- | --- |
|  | **Dietary pattern 1** | | | | **Dietary pattern 2** | | | | **Dietary pattern 3** | | | |
|  | T1  N=4124 | T2  N=4137 | T3  N=4142 | P-value | T1  N=4137 | T2  N=4133 | T3  N=4133 | P-value | T1  N=4134 | T2  N=4134 | T3  N=4135 | P-value |
| **Macronutrients** | | | | | | | | | | | | |
| Energy intake(kcal) | 3299.88±1218.90 | 2547.61±1074.19 | 3318.90±1166.42 | <001 | 2377.01±1138.96 | 2936.25±998.75 | 3853.35±990.24 | <001 | 2598.26±1124.58 | 2828.08±1085.71 | 3739.44±1102.15 | <001 |
| Protein(gr/day) | 114.11±46.84 | 95.52±38.05 | 122.60±51.14 | <001 | 82.07±35.24 | 107.84±38.86 | 140.30±46.17 | <001 | 105.99±52.06 | 103.80±43.25 | 121.62±42.33 | <001 |
| Carbohydrates(gr/day) | 537.31±224.17 | 381.06±185.36 | 429.89±165.53 | <001 | 332.14±170.32 | 446.14±186.81 | 570.00±185.84 | <001 | 361.02±171.73 | 428.57±192.26 | 567.51±196.25 | <001 |
| Fiber(gr/day) | 56.23±31.85 | 30.78±23.41 | 30.89±21.72 | <001 | 26.27±26.10 | 40.86±28.61 | 51.50±26.05 | <001 | 29.95±23.94 | 40.17±29.91 | 49.31±29.11 | <001 |
| Fat(gr/day) | 91.07±29.97 | 80.79±32.96 | 116.27±49.34 | <001 | 73.22±36.91 | 89.95±30.34 | 122.10±38.01 | <001 | 85.67±40.76 | 88.00±33.66 | 112.95±41.41 | <001 |
| SFAs(gr/day) | 26.74±8.55 | 25.30±10.79 | 35.46±16.31 | <001 | 22.88±11.85 | 27.40±10.04 | 37.24±12.93 | <001 | 27.47±14.61 | 26.54±10.92 | 33.50±12.46 | <001 |
| MUFAs(gr/day) | 33.10±11.54 | 26.62±12.54 | 40.12±21.73 | <001 | 26.42±17.76 | 31.11±12.29 | 42.34±15.94 | <001 | 28.55±16.03 | 30.10±12.80 | 41.20±18.39 | <001 |
| PUFAs(gr/day) | 19.73±8.27 | 18.17±9.27 | 32.75±22.63 | <001 | 20.25±17.30 | 21.06±11.84 | 29.36±17.47 | <001 | 20.62±14.55 | 20.46±12.32 | 29.58±19.43 | <001 |
| Cholesterol(gr/day) | 255.00±202.83 | 294.00±155.00 | 414.39±351.51 | <001 | 238.93±170.25 | 298.59±212.09 | 413.06±323.37 | <001 | 335.45±277.81 | 290.83±258.16 | 326.32±219.87 | <001 |
| **Micronutrients** | | | | | | | | | | | | |
| Vitamin B1(gr/day) | 3.51±1.79 | 2.33±1.18 | 2.38±0.94 | <001 | 2.00±1.25 | 2.82±1.47 | 3.43±1.33 | <001 | 2.27±1.18 | 2.76±1.52 | 3.26±1.54 | <001 |
| Vitamin B2(gr/day) | 2.45±1.03 | 2.06±0.89 | 2.55±0.98 | <001 | 1.72±0.73 | 2.29±0.83 | 3.02±0.94 | <001 | 2.18±1.01 | 2.26±0.96 | 2.62±0.95 | <001 |
| Vitamin B6(gr/day) | 2.47±0.98 | 1.94±0.83 | 2.74±1.37 | <001 | 1.79±1.11 | 2.27±0.85 | 3.03±1.03 | <001 | 2.22±1.17 | 2.24±1.11 | 2.66±1.01 | <001 |
| Vitamin C(gr/day) | 102.23±59.74 | 136.92±76.44 | 241.06±191.45 | <001 | 108.19±65.60 | 138.37±97.98 | 222.97±179.98 | <001 | 196.79±178.43 | 134.16±94.94 | 138.50±99.18 | <001 |
| Vitamin A(gr/day) | 438.24±251.54 | 294.82±410.62 | 106.86±302.94 | <001 | 98.89±161.72 | 288.34±280.07 | 470.78±453.72 | <001 | 151.61±294.03 | 307.62±354.89 | 408.46±367.09 | <001 |
| Vitamin E(gr/day) | 19.34±10.02 | 12.83±9.39 | 12.06±10.65 | <001 | 9.33±6.40 | 14.48±8.16 | 20.64±12.71 | <001 | 11.29±9.22 | 14.88±9.44 | 18.58±11.61 | <001 |
| Calcium(gr/day) | 1112.85±447.44 | 963.31±467.88 | 1081.66±491.11 | <001 | 735.12±290.67 | 1021.22±359.76 | 1392.18±491.47 | <001 | 971.25±480.90 | 1021.95±449.82 | 1167.37±465.79 | <001 |
| Magnesium(gr/day) | 750.60±395.17 | 428.45±310.04 | 400.81±209.14 | <001 | 339.46±291.99 | 550.19±247.90 | 701.38±327.59 | <001 | 385.14±271.65 | 533.24±358.85 | 685.03±365.54 | <001 |
| Sodium(gr/day) | 5883.50±2628.27 | 4917.48±2477.08 | 5567.08±4970.81 | <001 | 4718.49±4237.02 | 5313.95±2618.75 | 6335.13±3480.98 | <001 | 4612.22±3089.20 | 5319.77±2726.18 | 6434.63±4427.69 | <001 |
| Potassium(gr/day) | 4337.06±1538.01 | 3636.23±1595.12 | 4848.78±2113.54 | <001 | 2984.13±1217.80 | 4019.28±1167.93 | 5820.41±1776.30 | <001 | 4059.17±2121.06 | 3936.43±1591.88 | 4826.84±1619.99 | <001 |

Abbreviations. PCA: Principle Component Analysis; SFA: Saturated Fatty Acid; MUFA: Mono Unsaturated Fatty Acids; PUFA: Poly Unsaturated Fatty Acid

| **Supplementary table 2.** Age, sex, and energy-adjusted dietary of macronutrients and micronutrients of study participants according to tertiles of dietary patterns derived by PLS. | | | | | | | | | | | | |
| --- | --- | --- | --- | --- | --- | --- | --- | --- | --- | --- | --- | --- |
|  | **Dietary pattern 1** | | | | **Dietary pattern 2** | | | | **Dietary pattern 3** | | | |
|  | T1  N=4130 | T2  N=4134 | T3  N=4139 | P-value | T1  N=4143 | T2  N=4136 | T3  N=4124 | P-value | T1  N=4135 | T2  N=4132 | T3  N=4136 | P-value |
| **Macronutrients** | | | | | | | | | | | | |
| Energy intake(kcal) | 2480.66±1174.30 | 2878.78±1025.84 | 3805.04±1015.89 | <001 | 2802.35±1263.88 | 2641.22±919.07 | 3724.74±1123.18 | <001 | 2779.20±1155.63 | 2715.95±1086.37 | 3670.40±1140.73 | <001 |
| Protein(gr/day) | 89.87±42.95 | 105.47±35.79 | 138.85±47.99 | <001 | 102.00±49.44 | 98.46±39.48 | 129.28±45.10 | <001 | 107.20±51.45 | 99.89±40.34 | 124.15±44.71 | <001 |
| Carbohydrates(gr/day) | 396.66±220.12 | 440.68±195.27 | 521.94±176.41 | <001 | 345.39±150.83 | 394.69±151.53 | 598.36±209.33 | <001 | 394.03±176.36 | 405.37±190.00 | 554.28±208.95 | <001 |
| Fiber(gr/day) | 39.06±33.00 | 38.47±27.59 | 41.64±24.83 | <001 | 22.67±19.04 | 33.36±20.02 | 60.95±30.54 | <001 | 32.46±23.52 | 36.17±28.45 | 50.60±30.93 | <001 |
| Fat(gr/day) | 69.91±25.01 | 89.10±26.17 | 130.91±43.09 | <001 | 96.13±51.53 | 83.44±34.07 | 106.08±31.13 | <001 | 89.78±41.51 | 85.40±34.09 | 110.93±41.18 | <001 |
| SFAs(gr/day) | 20.86±6.76 | 27.03±8.16 | 39.60±14.88 | <001 | 30.32±16.68 | 25.83±11.13 | 31.36±9.83 | <001 | 27.55±13.44 | 26.09±10.89 | 33.87±13.52 | <001 |
| MUFAs(gr/day) | 24.53±10.63 | 30.01±10.85 | 45.28±19.08 | <001 | 34.36±22.82 | 27.79±12.01 | 37.72±11.73 | <001 | 29.64±16.14 | 28.91±12.76 | 41.30±18.28 | <001 |
| PUFAs(gr/day) | 15.71±7.07 | 20.29±9.10 | 34.66±21.65 | <001 | 27.87±21.95 | 19.69±13.15 | 23.11±10.31 | <001 | 22.74±16.03 | 20.19±11.78 | 27.74±19.24 | <001 |
| Cholesterol(gr/day) | 214.48±91.48 | 295.65±116.77 | 458.77±393.22 | <001 | 351.41±325.66 | 300.63±204.14 | 303.46±222.97 | <001 | 328.91±280.49 | 284.24±170.82 | 339.81±293.31 | <001 |
| **Micronutrients** | | | | | | | | | | | | |
| Vitamin B1(gr/day) | 2.65±1.75 | 2.72±1.42 | 2.92±1.14 | <001 | 1.94±0.77 | 2.46±1.08 | 3.78±1.70 | <001 | 2.32±1.08 | 2.54±1.41 | 3.43±1.64 | <001 |
| Vitamin B2(gr/day) | 1.89±0.91 | 2.26±0.81 | 2.96±0.95 | <001 | 2.07±0.92 | 2.12±0.85 | 2.81±1.01 | <001 | 2.24±0.97 | 2.15±0.91 | 2.66±1.02 | <001 |
| Vitamin B6(gr/day) | 1.88±0.93 | 2.22±0.95 | 3.07±1.13 | <001 | 2.21±1.34 | 2.06±0.88 | 2.81±0.96 | <001 | 2.40±1.20 | 2.11±1.08 | 2.59±1.01 | <001 |
| Vitamin C(gr/day) | 87.36±41.55 | 138.46±65.48 | 256.32±188.94 | <001 | 176.98±148.69 | 148.04±124.86 | 147.25±125.02 | <001 | 233.41±187.59 | 126.63±72.55 | 109.99±63.91 | <001 |
| Vitamin A(gr/day) | 241.92±207.69 | 319.9±336.93 | 301.65±482.16 | <001 | 22.71±61.22 | 263.22±324.46 | 545.41±356.55 | <001 | 184.71±356.15 | 273.81±338.08 | 405.73±337.59 | <001 |
| Vitamin E(gr/day) | 13.37±8.35 | 14.82±10.22 | 16.59±12.64 | <001 | 8.74±7.40 | 13.53±8.89 | 21.59±10.60 | <001 | 11.42±8.62 | 13.79±9.27 | 19.46±11.83 | <001 |
| Calcium(gr/day) | 843.98±378.07 | 1037.44±402.20 | 1303.28±522.51 | <001 | 855.61±372.13 | 966.58±425.89 | 1309.16±483.01 | <001 | 993.74±448.33 | 981.82±451.24 | 1182.71±491.50 | <001 |
| Magnesium(gr/day) | 517.85±411.27 | 526.88±349.87 | 553.30±286.38 | <001 | 290.38±132.37 | 457.10±260.26 | 820.15±376.16 | <001 | 406.42±254.73 | 483.38±343.35 | 710.18±384.83 | <001 |
| Sodium(gr/day) | 4909.02±2613.17 | 5307.33±2629.86 | 6149.13±4868.70 | <001 | 5018.52±4649.66 | 4936.48±2817.79 | 6415.37±2701.93 | <001 | 4766.20±3170.72 | 5183.12±3073.34 | 6417.10±4158.52 | <001 |
| Potassium(gr/day) | 3195.90±1341.47 | 4012.78±1285.48 | 5611.23±1910.17 | <001 | 3800.60±1870.11 | 3814.12±1538.29 | 5211.37±1716.28 | <001 | 4543.85±2210.15 | 3727.68±1553.00 | 4550.58±1542.20 | <001 |

Abbreviations. PLS: Partial Least Square; SFA: Saturated Fatty Acid; MUFA: Mono Unsaturated Fatty Acids; PUFA: Poly Unsaturated Fatty Acid

| **Supplementary table 3.** Age, sex, and energy-adjusted dietary of macronutrients and micronutrients of study participants according to tertiles of dietary patterns derived by RRR. | | | | | | | | | | | | |
| --- | --- | --- | --- | --- | --- | --- | --- | --- | --- | --- | --- | --- |
|  | **Dietary pattern 1** | | | | **Dietary pattern 2** | | | | **Dietary pattern 3** | | | |
|  | T1  N=4133 | T2  N=4122 | T3  N=3631 | P-value | T1  N=4098 | T2  N=4109 | T3  N=3679 | P-value | T1  N=4138 | T2  N=4136 | T3  N=4129 | P-value |
| **Macronutrients** | | | | | | | | | | | | |
| Energy intake(kcal) | 2204.86±971.70 | 2989.83±977.41 | 3970.32±960.01 | <001 | 3557.43±1179.25 | 2904.42±1044.78 | 2906.16±1227.90 | <001 | 2939.53±1147.70 | 2685.52±1073.06 | 3541.78±1237.74 | <001 |
| Protein(gr/day) | 82.15± 36.22 | 108.84± 36.05 | 143.77± 46.64 | <001 | 123.88±46.20 | 98.72±40.76 | 107.88±49.80 | <001 | 115.85±5104 | 96.83±38.69 | 118.34±47.02 | <001 |
| Carbohydrates(gr/day) | 344.22± 185.32 | 460.25± 192.40 | 559.30± 179.27 | <001 | 576.02±214.01 | 409.86±175.44 | 354.99±149.47 | <001 | 389.74±161.60 | 397.55±176.37 | 561.84±223.83 | <001 |
| Fiber(gr/day) | 31.24±28.41 | 41.96±29.16 | 46.58±26.46 | <001 | 59.21±30.49 | 35.04±22.63 | 22.99±18.87 | <001 | 28.66±20.45 | 33.96±24.67 | 56.01±32.31 | <001 |
| Fat(gr/day) | 64.87± 21.17 | 92.32± 24.54 | 132.92± 41.40 | <001 | 99.32±32.06 | 83.07±33.06 | 104.09±51.85 | <001 | 101.89±46.25 | 84.11±35.74 | 99.88±36.77 | <001 |
| SFAs(gr/day) | 19.47±5.71 | 27.68±7.89 | 40.34±14.15 | <001 | 29.23±9.68 | 25.41±10.39 | 32.86±16.93 | <001 | 32.79±16.39 | 25.69±10.54 | 29.03±10.55 | <001 |
| MUFAs(gr/day) | 21.99±8.86 | 31.42±10.20 | 46.43±19.17 | <001 | 34.67±12.19 | 28.09±12.29 | 37.10±22.59 | <001 | 33.97±18.40 | 28.73±14.41 | 37.16±16.49 | <001 |
| PUFAs(gr/day) | 15.01±7.04 | 20.94±9.07 | 33.28±16.87 | <001 | 22.11±10.97 | 19.34±11.05 | 29.22±22.37 | <001 | 25.76±18.36 | 20.67±13.86 | 24.24±15.90 | <001 |
| Cholesterol(gr/day) | 216.26± 93.48 | 294.06± 122.18 | 458.32± 390.32 | <001 | 278.82±140.97 | 281.24±149.11 | 400.07±390.14 | <001 | 397.46±329.52 | 283.16±165.98 | 273.95±225.04 | <001 |
| **Micronutrients** | | | | | | | | | | | | |
| Vitamin B1(gr/day) | 2.27±1.49 | 2.87±1.51 | 3.18±1.23 | <001 | 3.59±1.71 | 2.57±1.26 | 2.04±0.82 | <001 | 2.29±0.93 | 2.42±1.21 | 3.541.80 | <001 |
| Vitamin B2(gr/day) | 1.72±0.75 | 2.34±0.79 | 3.08±0.93 | <001 | 2.71±1.02 | 2.11±0.87 | 2.21±0.97 | <001 | 2.44±1.02 | 2.10±0.87 | 2.51±1.03 | <001 |
| Vitamin B6(gr/day) | 1.71±0.90 | 2.32±0.87 | 3.16±1.08 | <001 | 2.82±1.04 | 1.06±0.82 | 2.21±1.31 | <001 | 2.53±1.27 | 2.05±1.01 | 2.52±0.99 | <001 |
| Vitamin C(gr/day) | 92.97±49.05 | 136.60±69.04 | 251.87±189.56 | <001 | 188.17±178.93 | 129.60±93.87 | 151.73±100.07 | <001 | 238.88±187.82 | 129.06±72.41 | 104.98±59.79 | <001 |
| Vitamin A(gr/day) | 173.81±190.40 | 325.13±312.49 | 373.39±488.09 | <001 | 510.68±386.40 | 272.55±302.06 | 54.81±174.74 | <001 | 108.57±308.42 | 307.62±395.04 | 439.52±269.09 | <001 |
| Vitamin E(gr/day) | 11.20±7.25 | 15.38±9.60 | 18.41±13.05 | <001 | 19.27±9.90 | 14.52±10.45 | 10.31±9.21 | <001 | 11.14±9.35 | 13.45±9.15 | 19.86±10.99 | <001 |
| Calcium(gr/day) | 761.26±318.36 | 1057.06±372.92 | 1374.86±507.82 | <001 | 1268.09±478.13 | 962.51±414.98 | 908.95±438.88 | <001 | 1061.73±512.65 | 972.48±442.84 | 1120.90±449.41 | <001 |
| Magnesium(gr/day) | 414.98±353.96 | 566.34±362.93 | 625.56±310.51 | <001 | 776.66±384.67 | 484.45±299.78 | 311.94±161.20 | <001 | 369.21±193.01 | 454.20±301.08 | 767.58±402.44 | <001 |
| Sodium(gr/day) | 4543.33±2499.48 | 5427.90±2942.34 | 6394.70±4651.64 | <001 | 6174.74±2995.28 | 4974.47±2640.95 | 5216.67±4635.24 | <001 | 4784.10±3390.04 | 5194.54±3331.20 | 6390.12±3780.57 | <001 |
| Potassium(gr/day) | 2886.98±1091.94 | 4102.71±1138.69 | 5831.24±1803.80 | <001 | 5383.65±1885.25 | 3730.56±1386.14 | 3713.32±1676.79 | <001 | 4591.37±2165.51 | 3723.49±1582.31 | 4507.96±1571.36 | <001 |

Abbreviations. RRR: Reduced Rank Regression; SFA: Saturated Fatty Acid; MUFA: Mono Unsaturated Fatty Acids; PUFA: Poly Unsaturated Fatty Acid
